# *Meeting report*: Fifth Italo-Hungarian Symposium on Spectrochemistry Quality Control and Assurance in Life Sciences

**DOI:** 10.1155/S1463924692000063

**Published:** 1992

**Authors:** Sergio Caroli, Roger Fuoco

**Affiliations:** 1 Istituto Superiore di Sanità Rome Italy; 2 Istituto di Chimica Analitica Strumentale CNR Pisa Italy


Journal of Automatic Chemistry, Vol. 14, No. 1 (January–February 1992), p. 28

*Meeting report*

### Fifth Italo–Hungarian Symposium on Spectrochemistry Quality Control and Assurance in Life Sciences

The fifth Italo–Hungarian Symposium was held in Pisa from 9–13 September 1991, and was jointly organized, as part of a bilateral scientific and technological agreement, by the National Institute of Health in Rome and the Institute of Instrumental Analytical Chemistry, National Council of Research in Pisa. The opening ceremony was held in the Congress Centre in Pisa, followed by a reception in the City Hall (Sala della Baleari), which was hosted by the town authorities. The symposium was dedicated to Professor Károly Zimmer, who died three weeks before it, in recognition of his work for the bilateral co-operation programme.

The five-day Symposium covered the various aspects of spectrochemistry, with special emphasis on the concepts and criteria for Quality Control (QC) and Quality Assurance (QA) of analytical measurements. Such topics as the proper planning of investigations, the adoption of good laboratory practice principles, the preparation of new reference materials and their correct use, and the sound assessment of reference values for elements and organic substances in a diversity of matrices were discussed.

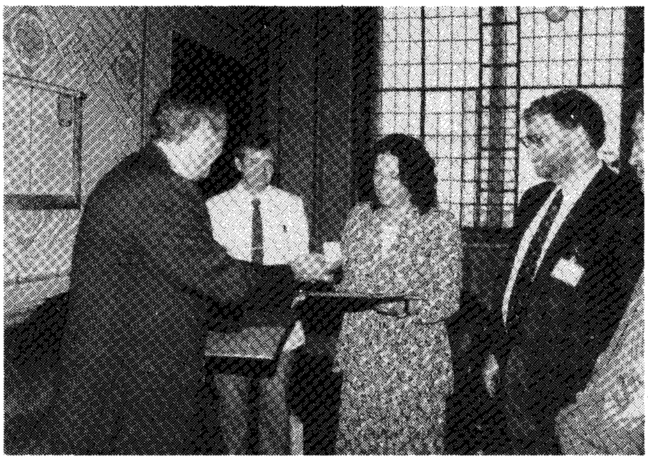

*Presentation to representatives of international organizations by the Mayor of Pisa at the Fifth Italo–Hungarian Symposium on Spectrochemistry Quality Control and Quality Assurance in the Life Sciences (September 1991).*

There were 36 lectures and 36 poster presentations. The 14 sessions included general aspects of QC-QA, QC-QA in environmental studies; Advances in instrumentation and applications; QC-QA in biomedical studies; QC-QA in industry and the environment, Progress in chromatography, The manufacturers' viewpoint; QC-QA in the food industry.

As well as scientists from Italian and Hungarian institutions, a number of speakers from other countries also attended the Symposium, including J.-J. Belliardo (EC); S. Cerjan-Stefanović (Yugoslavia); K. Grob (Switzerland); P. R. Griffiths (USA); W. Wegscheider (Austria) and W. Zyrnicki (Poland).

The Symposium was intended, and clearly succeeded, to present the international state-of-the-art in spectrochemical QA and QC.

The sixth meeting is being planned for June 1992 in Hungary and will probably have the official support of Austria, Czechoslovakia, Yugoslavia and Poland.

**Sergio Caroli and Roger Fuoco**
*Istituto Superiore di Sanità, Rome; and Istituto di Chimica Analitica Strumentale, CNR, Pisa, Italy*

0142–0453/92 $3.00 © 1992 Taylor & Francis Ltd.

